# Planetary health in practice: A scoping review of education and training for nurses and midwives

**DOI:** 10.1016/j.ijnsa.2026.100670

**Published:** 2026-09-10

**Authors:** Sadia Afrin, Pascal Petit, Nicolas Vuillerme, Muhammad Asaduzzaman

**Affiliations:** a Department of Public Health, North South University, Dhaka, 1229, Bangladesh; b Université Grenoble Alpes, CNRS, Grenoble INP, LIG, SANGRIA, Grenoble, France; c Institut Universitaire de France, Paris, France; d Department of Community Medicine and Global Health, Institute of Health and Society, Faculty of Medicine, University of Oslo, Oslo, Norway; e Board Member, American Society of Tropical Medicine and Hygiene (ASTMH), Arlington, VA, 22202, United States of America

**Keywords:** Planetary health education, Midwifery, Nurse, Maternal-child health services, Staff development, Clinical competence, Scoping review

## Abstract

**Background:**

Planetary health is an emerging field of growing global importance. Environmental changes increasingly threaten maternal and child health, while nurses and midwives play central roles across care processes. Despite these opportunities, planetary health education and training in nursing and midwifery are underdeveloped and a mapping of current approaches is critically needed.

**Objective:**

To map existing planetary health education and training initiatives for nurses and midwives and examine their reported educational and practice outcomes related to maternal and child health.

**Methods:**

A scoping review was conducted following the Arksey and O'Malley framework Searches were conducted in Medline/PubMed, Scopus, and EMBASE for English-language studies published from 1 January 2015 to 31 January 2026. Eligible studies addressed planetary health education and/or training for nurses or midwives. We used a standardized form to extract data, and narrative synthesis was applied given the diversity of interventions and outcomes.

**Results:**

Out of 258 records identified, seven studies published from 2019 to 2026 met the inclusion criteria. These were conducted in Australia(n = 1), Australia and New Zealand (n = 1), Spain (n = 1), Turkey (n = 2), and Uganda (n = 2). The evidence showed marked heterogeneity in study design, settings, and educational approaches. Study designs included two randomized controlled trials, one quasi-experimental study, one cross-sectional survey, two qualitative studies, and one implementation study. Educational approaches varied widely, from online courses and curriculum validation to community-based biomass smoke education and simulation-based training. Reported outcomes were grouped into five categories: knowledge, attitudes, confidence and self-efficacy, behavioral practice change, and clinical or system-level outcomes. Five studies reported improvements in attitude and satisfaction. Confidence and self-efficacy outcomes were infrequently reported. Behavioral or practice change was noted in three studies. Two studies reported direct maternal and child health benefits through reduced household air pollution exposure, but no study reported direct maternal or neonatal clinical outcomes.

**Conclusion:**

Evidence on planetary health education for nurses and midwives is emerging but remains limited. Current studies focus mainly on immediate educational outcomes rather than sustained practice change or patient-level impact. Future research should evaluate long-term competency development, clinical practice change, and measurable maternal, neonatal, and child health outcomes.

**Registration:**

OSF | Planetary Health Education and Training for Midwives and Nurses: A Scoping Review of Reported Educational and Practice Outcomes Related to Maternal and Child Health, registered 5/2/2026, https://doi.org/10.17605/OSF.IO/JU3GY

**Social media abstract:**

Current planetary health education and interventions for nurses and midwives have improved knowledge, attitudes, and some practice-related behaviors but lack direct patient-level outcomes. @zamansohag08

**What is already known**

• Planetary health risks pose serious threats to maternal and child health.
• Nurses and midwives are the largest workforce to address planetary health determinants of maternal and child health.
• A lack of standardized planetary health competencies is evident in nursing and midwifery curricula.

**What this paper adds**

• Our review maps existing global planetary health education and training for nurses and midwives.
• This review found that current planetary health education & interventions lack direct patient-level outcomes.
• Planetary health education for nurses and midwives improves knowledge, attitudes and practice-related behaviors.

## 1 Background

People and the planet where they live are interlinked with an inextricable tie. Our surrounding ecosystem and natural world are altering rapidly due to global warming, biodiversity loss, land degradation, ambient air pollution and degradation of freshwater systems (Romanello et al., 2023; Whitmee et al., 2015; Watts et al., 2019). The Rockefeller Foundation–Lancet Commission defined planetary health as safeguarding human civilization in the Anthropocene and warned that environmental destabilization threatens decades of global health progress (Whitmee et al., 2015). The principles of planetary health recognize the interdependence between human health and natural systems, including climate, biodiversity, air, water, food systems, and ecosystems (Jochem et al., 2023; Whitmee et al., 2015). These principles therefore call for systems thinking, health equity, intergenerational responsibility, climate resilience, prevention of environmental harm, and sustainable healthcare delivery (World Health Organization, 2023). Recent Lancet Countdown reports further reinforce the urgency of this perspective, highlighting human-induced climate change as an escalating systemic threat to public health and health systems worldwide (Watts et al., 2019; Romanello et al., 2023).

Among all health system indicators, maternal and child health is a vital cornerstone which lies at the intersection of planetary disruption and intergenerational equity. Climate change contributes to multiple environmental exposures (Barouki, 2024) during pregnancy, which represents a critical window of vulnerability for both the mother and the child and may have lasting effects on their health (Reali and De Guchtenaere, 2025; Rylander et al., 2013; Sheffield and Landrigan, 2010). Premature birth, low birth weight and stillbirth are associated with exposure to air pollutants such as fine particulate matter (PM2.5) (Bekkar et al., 2020; Sun et al., 2015). Similarly elevated heat exposure during pregnancy has been linked to preeclampsia and preterm birth (Chersich et al., 2018; Zhang et al., 2017). Beyond air pollution and heat exposure, planetary health risks also include chemical and toxicological exposures, such as endocrine-disruption chemicals, pesticides, and other pollutants that may affect reproductive, fetal, and child development pathways (Barouki, 2024; Sheffield and Landrigan, 2010). Including these exposures is important because planetary health education should prepare nurses and midwives to recognize a broader range of environmental determinants of maternal and child health, not only climate-related hazards. Climate-driven food and water insecurity can further worsen maternal malnutrition, infectious disease transmission, and impaired child development, disproportionately affecting populations in low- and middle-income countries, with the least capacity in health systems to adapt (Reali and De Guchtenaere, 2025; Rylander et al., 2013; Sheffield and Landrigan, 2010). These intersecting factors all exacerbate existing health inequities and jeopardize efforts to reduce maternal and neonatal mortality worldwide (Rylander et al., 2013; Sheffield and Landrigan, 2010).

Therefore, health systems are increasingly urged to become climate-resilient and environmentally sustainable (Romanello et al., 2023; World Health Organization, 2023). Such resilience depends fundamentally on workforce preparedness who will be capable of addressing the challenges of planetary disruption (Frenk et al., 2010). Nurses and midwives constitute the largest segment of the global health workforce and are central to antenatal, intrapartum, and postnatal care delivery (World Health Organization, 2020). Across community and facility settings, they are uniquely positioned to identify environmental exposures, to provide risk assessment, health education, early detection of complications, continuity of care, counsel families on mitigation strategies, and support climate-informed clinical practice(Streater and Walton, 2025; World Health Organization, 2020). However, these responsibilities require knowledge and skills beyond traditional clinical training-such as climate literacy, understanding of environmental determinants of health (e.g., pregnancy outcomes), effective risk communication, and a capacity for advocacy for systems-level sustainability (Jochem et al., 2023; Frenk et al., 2010; Wellbery et al., 2018).

The Call to integrate climate change and planetary health into health professional education has intensified over the past decade (Maxwell and Blashki, 2016; Wellbery et al., 2018). Planetary health education refers to education and training that prepares nurses, midwives, students, and educators to recognize environmental determinants of health, communicate climate- and environment-related risks, and apply sustainable practice in maternal, newborn, and child health care. Medical education has responded relatively quickly to this call, with planetary health content increasingly embedded into core curricula, competency frameworks, and accreditation standards across medical schools (Wellbery et al., 2018). By contrast nursing and midwifery education has received comparatively limited systematic attention with reported initiatives largely confined to isolated modules, elective content, or single-institution pilots rather than embedded, system-wide curricular reform (Dupraz and Burnand, 2021; Attawet et al., 2025; Ganapathy et al., 2024; Portela Dos Santos et al., 2023; Salvador Costa et al., 2023). This is a notable gap, since nurses and midwives represent the largest share of the global health workforce and are central to maternal and child health service delivery, as outlined above. Emerging initiatives highlight the need to equip nurses and midwives with competencies in climate science, environmental risk communication, sustainable healthcare delivery, and disaster preparedness (Maxwell and Blashki, 2016; Wellbery et al., 2018; World Health Organization, 2021).

Understanding the scope of this gap requires clarity regarding the underlying constructs that these educational initiatives aim to develop. Planetary health education frameworks include systems thinking, environmental determinants of health, and workforce roles in mitigation and adaptation, and emphasize environmental justice and communication as core competencies (Guzmán et al., 2021; Jacobsen et al., 2024). Nonetheless, mere inclusion of content in the curriculum is insufficient; transitioning planetary health principles into enduring professional practice requires tangible improvements in knowledge, shifts in attitude, enhanced self-efficacy, and demonstrable changes in clinical behaviors. Sustained practice change further depends on institutional reinforcement and integration into policy and accreditation frameworks (World Health Organization, 2021; Frenk et al., 2010). However, the scale, consistency, and evaluative rigor of these educational efforts have not been systematically mapped, and it remains unclear whether planetary health education for nurses and midwives translates into substantive clinical adaptation or remains limited to awareness. Existing evidence in this field is fragmented across disciplines and professional groups; studies on environmental health education rarely report outcomes related to maternal health, and assessments of climate change awareness rarely make distinctions based on profession (Wellbery et al., 2018; Maxwell and Blashki, 2016). Although a recent review Attawet et al. (2025) examined midwives’ preparedness for the impact of climate change on maternal and child health, its focus was limited to preparedness in relation to specific risks, particularly extreme heat and air pollution, rather than the wider field of planetary health education and training across nursing and midwifery. Previous reviews have otherwise frequently focused on physicians or medical students (Wellbery et al., 2018), leaving a gap in synthesized evidence about nurses and midwives-despite their critical roles throughout the maternal and child health continuum of care (Wellbery et al., 2018; World Health Organization, 2021). In addition, the global distribution of planetary health education efforts, especially in low- and middle-income countries where maternal and planetary health vulnerabilities are most severe, has received little attention (Reali and De Guchtenaere, 2025; Rylander et al., 2013).

Therefore, this scoping review aimed to identify and synthesize planetary health-related education and training initiatives within nursing and midwifery contexts, including programmed involving nurses, midwives, nursing and midwifery students, and educators. It also aimed to describe the reported outcomes of these initiatives across knowledge, attitudes or acceptability, confidence or self-efficacy, practice-related changes, curriculum or system-level outcomes, and maternal and child health relevance.

## 2 Methods

We carried out a scoping review following the Arksey and O’Malley (2005) methodology and reported using the Preferred Reporting Items for Systematic reviews and Meta-Analyses extension for Scoping Reviews guidelines (Tricco et al., 2018). The Arksey and O’Malley framework provides a structured approach to systematically identify, chart and categorize existing literature to describe key concepts and evidence gaps without formally assessing study quality or confidence in our findings. A scoping review was considered the most appropriate review type for exploring the diverse body of literature given the novel area of research and the study questions. Our scoping review is registered on the open science framework (https://osf.io/ju3gy).

### 2.1 Search strategy

A broad search strategy was developed by the review team in consultation with a search expert and was structured using the Population–Concept–Context framework. The population included nurses, midwives, nursing students, midwifery students, and nursing/midwifery educators. The concept was planetary health-related education or training within nursing and midwifery contexts, and the context included academic, clinical, community, primary care, and maternal and child health settings. Search terms included combinations of keywords such as “midwives”, “nurse”, “education”, “planetary health”, etc. combined with Boolean Operators to search different databases including Medline/PubMed, Scopus, and EMBASE. Additionally, a manual search for citations of relevant systematic reviews was performed to locate additional relevant studies. The search timeframe was set from 1st January 2015 to 31st January 2026, reflecting the launch of the concept by the Rockefeller Foundation–Lancet Commission on planetary health in 2015 (Whitmee et al., 2015). **Supplementary Table 1** presents the key terms for developing the comprehensive search strategy and **Supplementary Table 2** presents the comprehensive search strategies developed for PubMed, EMBASE, and Scopus, including the number of records retrieved from each database.

### 2.2 Eligibility criteria

Eligibility criteria were defined using the Population, Concept, Context framework, together with study design and language of restrictions.

#### 2.2.1 Inclusion criteria

Studies were eligible if they met all the following criteria:

(i) Population: nurses, midwives, nursing students, midwifery students, or nursing and midwifery educators.
(ii) Concept: planetary health education and/or training, including undergraduate curricula or broader university-level planetary health initiatives with a clear nursing or midwifery education component or outcomes relevant to the review population.
(iii) Context: nursing and midwifery education or practice settings, including academic, clinical, community, or primary care settings, where relevant to maternal, newborn, or child health.

Primary research studies of any design were eligible, as were opinion-based publications (e.g., viewpoints, perspectives, editorials) that met these criteria. Only English-language publications were included.

#### 2.2.2 Exclusion criteria

Studies were excluded if they:

(i) Addressed planetary health or sustainability content without an identifiable education, training, or curriculum-development component.
(ii) Reported on populations entirely outside nursing and midwifery, with no distinguishable nursing or midwifery-specific data.
(iii) Were conference abstracts, protocols, or theses without extractable outcome data.
(iv) Lacked direct or indirect relevance to maternal, newborn, or child health.

### 2.3 Study selection

The search results were imported into the Rayyan software (online) developed by Qatar Computing Research Institute (Ouzzani et al., 2016). After removing duplicates, two independent reviewers (SA and PP) screened titles and abstracts. The full texts of potentially eligible articles were then screened independently by the same two reviewers (SA and PP), following the prioritization and sequential exclusion method (Saif-Ur-Rahman et al., 2022). Under this approach, the Population, Concept, Context eligibility criteria were applied in a fixed sequence (population, then concept, then context) during full-text review, and each article was excluded at the first criterion it failed to meet, with the corresponding reason recorded; articles satisfying all criteria in sequence were retained for data extraction. Any disagreements during title and abstract or full-text screening were first discussed between the two reviewers. If consensus could not be reached, a third reviewer (MA) made the final decision.

### 2.4 Data extraction

A standard Excel-based data extraction form was developed, reviewed by the research team, and piloted on two eligible studies before full implementation to ensure consistency and data accuracy. The form used predefined, standardized fields and closed response categories for each outcome domain (knowledge, attitudes/acceptability, confidence/self-efficacy, practice-related outcomes, and curriculum/system-level outcomes), operationalized according to the definitions below, which limited free-text entry and reduced variation between reviewers in how findings were classified and recorded.

Two reviewers independently (SA and PP) extracted data from each selected article. Any discrepancies between the two reviewers were first discussed and checked against the full text of the included articles. Differences were resolved through consensus, and if agreement could not be reached, a third reviewer (MA) was consulted to make the final decision. After consensus was reached, the final extracted data were compiled using the Excel-based extraction form. The extraction process involved collecting information on the study context, objectives, methodology, educational curriculum, training program and key findings. Outcome measures were operationalized prior to synthesis and defined as follows. Knowledge referred to participants’ awareness, understanding, factual knowledge, test scores, or perceived knowledge of planetary health, climate change, sustainability, environmental health, and environmental determinants relevant to maternal and child health. Attitudes referred to participants’ views, perceptions, acceptability, motivation, satisfaction, or perceived importance of planetary health education or curriculum integration. Confidence or self-efficacy referred to participants’ perceived ability to explain, teach, counsel, communicate, or apply planetary health concepts in nursing, midwifery, education, or maternal and child health-related practice. Clinical or practice-related outcomes referred to reported or observed changes in professional behavior, educational practice, counselling practice, sustainable healthcare practice, or community-based maternal health practices, such as exposure reduction or improved household practices. Maternal and child health relevance was defined as either direct relevance, where studies addressed pregnancy, breastfeeding, maternal nutrition, infant health, or household exposures affecting mothers and children, or indirect relevance, where studies addressed workforce preparedness, student competencies, educator capacity, or curriculum integration relevant to future maternal and child health care.

### 2.5 Data synthesis

A narrative synthesis approach was used, following the guidance proposed by Popay et al. (2006). Findings were organized into predefined outcome domains: knowledge, attitudes or acceptability, confidence or self-efficacy, practice-related outcomes, system- or curriculum-level outcomes, and maternal and child health relevance. Outcomes within each domain were compared across studies to identify patterns, differences, and evidence gaps. As the included studies used different measurement tools and reporting formats, findings could not be statistically pooled and were instead compared descriptively across studies within each domain. The synthesis focused on mapping the available evidence and clarifying how planetary health education and training relates to nursing, midwifery, and maternal and child health practice. To support the narrative synthesis, visual representations including a Preferred Reporting Items for Systematic reviews and Meta-Analyses extension for Scoping Reviews flow diagram of the study selection process and summary evidence maps illustrating the distribution of included studies across outcome domains, geographic region, and study design were created using Microsoft PowerPoint and R version 4.5.2 (R Foundation for Statistical Computing, Vienna, Austria) on Windows 11 (Team, 2014), with both the tidyverse package version 2.0.0 (Wickham et al., 2019) and patchwork package version 1.3.2 (Pedersen, 2019).

## 3 Results

**Fig. 1:**
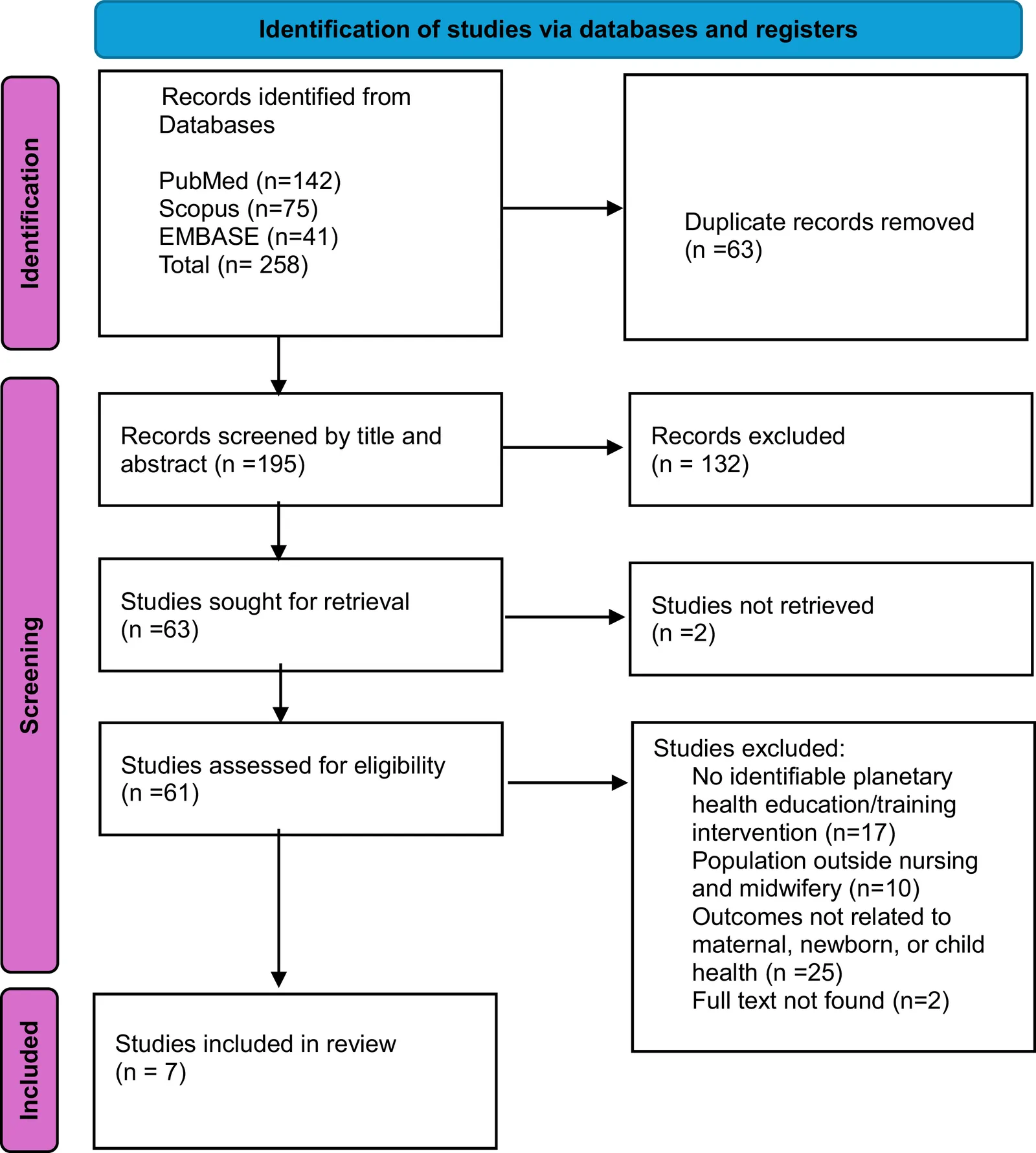
PRISMA flow diagram depicting the study selection process.

In total, we identified and screened 258 potential studies by conducting a comprehensive search in selected databases. Of the 61 studies assessed for full-text eligibility, 54 were excluded for not meeting the eligibility criteria. The reasons for exclusion were absence of an education, training, or curriculum development component (n = 17), ineligible study population (n = 10), outcomes not related to maternal and child health (n = 25), and unavailability of full text (n = 2). Finally, seven articles that met the inclusion criteria were selected for data extraction. Supplementary Table 3 presents these studies. All seven are peer reviewed primary research articles. Fig. 1 presents the Preferred Reporting Items for Systematic reviews and Meta-Analyses extension for Scoping Reviews flow diagram of the selection process.

### 3.1 Characteristics of the included studies

**Fig. 2:**
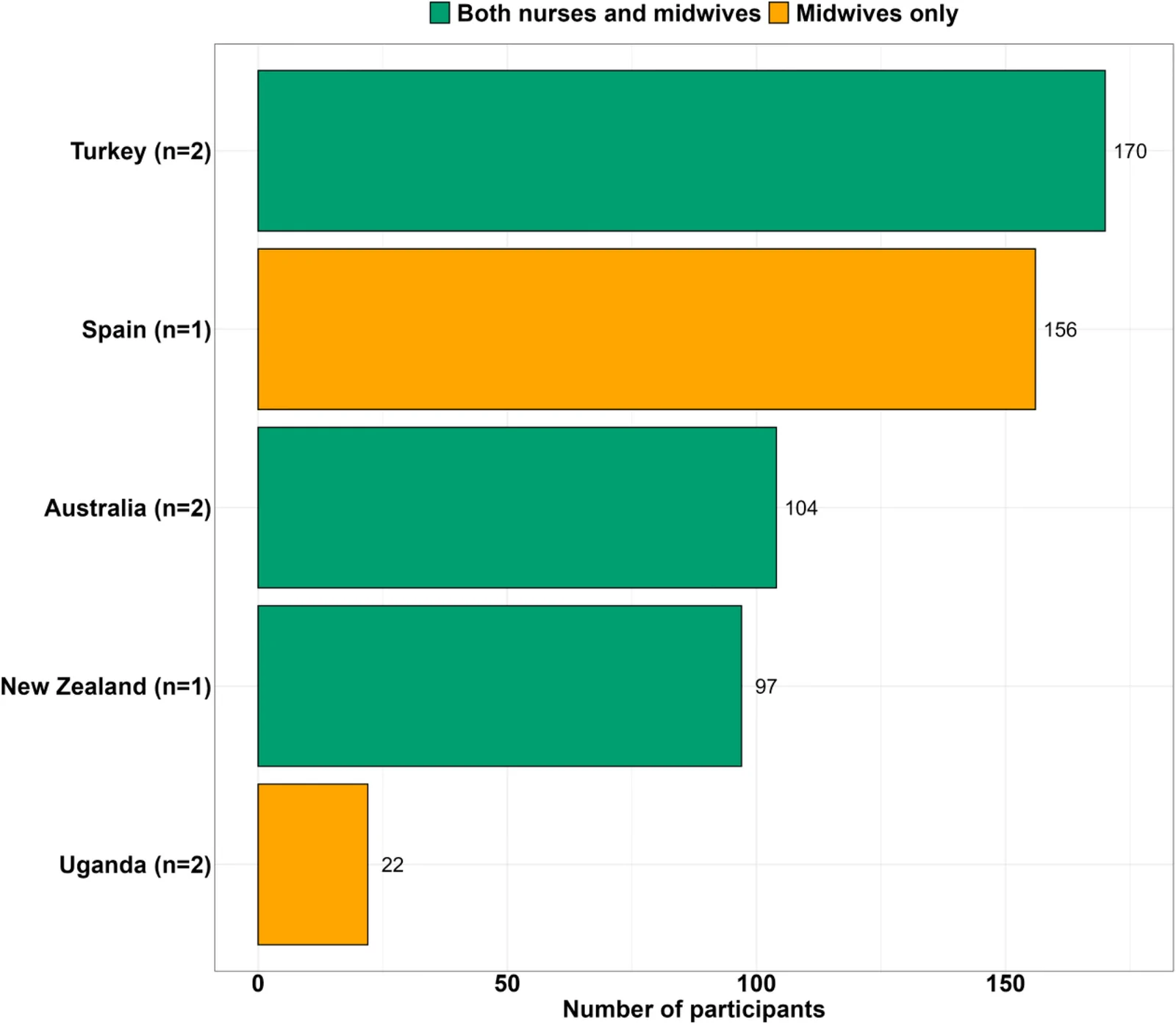
Geographic composition of included studies.

A summary of the seven included publications is presented in Table 1. All were published between 2019 and 2026. These studies were conducted in Turkey (İlaslan and Orak, 2025; Kırca and Daglı, 2025),Uganda (Cartwright et al., 2022; Nantanda et al., 2019), Spain (Cabedo-Ferreiro et al., 2026), (Catling et al., 2025) and or across Australia and New Zealand (Bonnamy et al., 2025) (Fig. 2).

**Table 1:** Characteristics of included studies.

**Author, year**	**Country**	**Participants**	**Sample size**	**Study Design**	**Education / Training Type**	**Aim**
Bonnamy et al., 2025	Australia, New Zealand	Nursing and midwifery educators	97	Cross-sectional study	Planetary health knowledge and teaching practices	To explore Australian and New Zealand nursing and midwifery educators' planetary health knowledge, views, confidence, and teaching practices.
Cabedo-Ferreiro et al., 2026	Spain	Midwives	156	Quasi-experimental study	Planetary health and sustainable nutrition training	To train healthcare professionals, predominantly midwives, in breastfeeding and sustainable, healthy nutrition to foster behavioral change toward sustainability among pregnant and breastfeeding women.
Catling et al., 2025	Australia	Midwifery students	7	Qualitative study	Planetary health curriculum development	To validate previously identified planetary health knowledge and skill statements and determine their appropriateness for undergraduate midwifery curricula.
İlaslan and Şahin Orak, 2025	Turkey	Nursing students	70	Randomized controlled trial	Sustainable healthcare education with simulation	To investigate the effects of sustainable healthcare education using cooperative learning and simulation on nursing students' sustainable healthcare knowledge, attitude, and skills, compared with self-directed learning.
Kırca and Daglı, 2025	Turkey	Midwifery students	100	Randomized controlled trial	Climate change education (Pecha-Kucha method)	To evaluate the impact of the Pecha-Kucha method versus PowerPoint presentation on midwifery students' climate change awareness and satisfaction.
Cartwright et al., 2022	Uganda	Midwives	10	Qualitative study	Biomass smoke health education program	To examine the longer-term impact and sustained feasibility of a midwife-led biomass smoke education program from the perspective of midwives, village health team members, and mothers.
Nantanda et al., 2019	Uganda	Midwives	12	Implementation study	Midwife-led biomass smoke education	To assess the feasibility, acceptability, and impact of a midwife-led education program on biomass smoke risks and prevention for women attending maternity clinics in Uganda.

Bar plot showing the total number of participants by country. Numbers in brackets next to each country indicate the number of studies conducted. Bar colors denote the type of healthcare professionals included: orange for midwives and green for studies including both nurses and midwives.

Two studies used randomized controlled trial designs, one used a cross-sectional survey design, one applied a quasi-experimental design, two employed qualitative approaches, and one was an implementation study. Educational approaches included curriculum development, educator survey work, online training, simulation-based teaching, the Pecha-Kucha method, and community-based biomass smoke education.

### 3.2 Synthesis of results

**Fig. 3:**
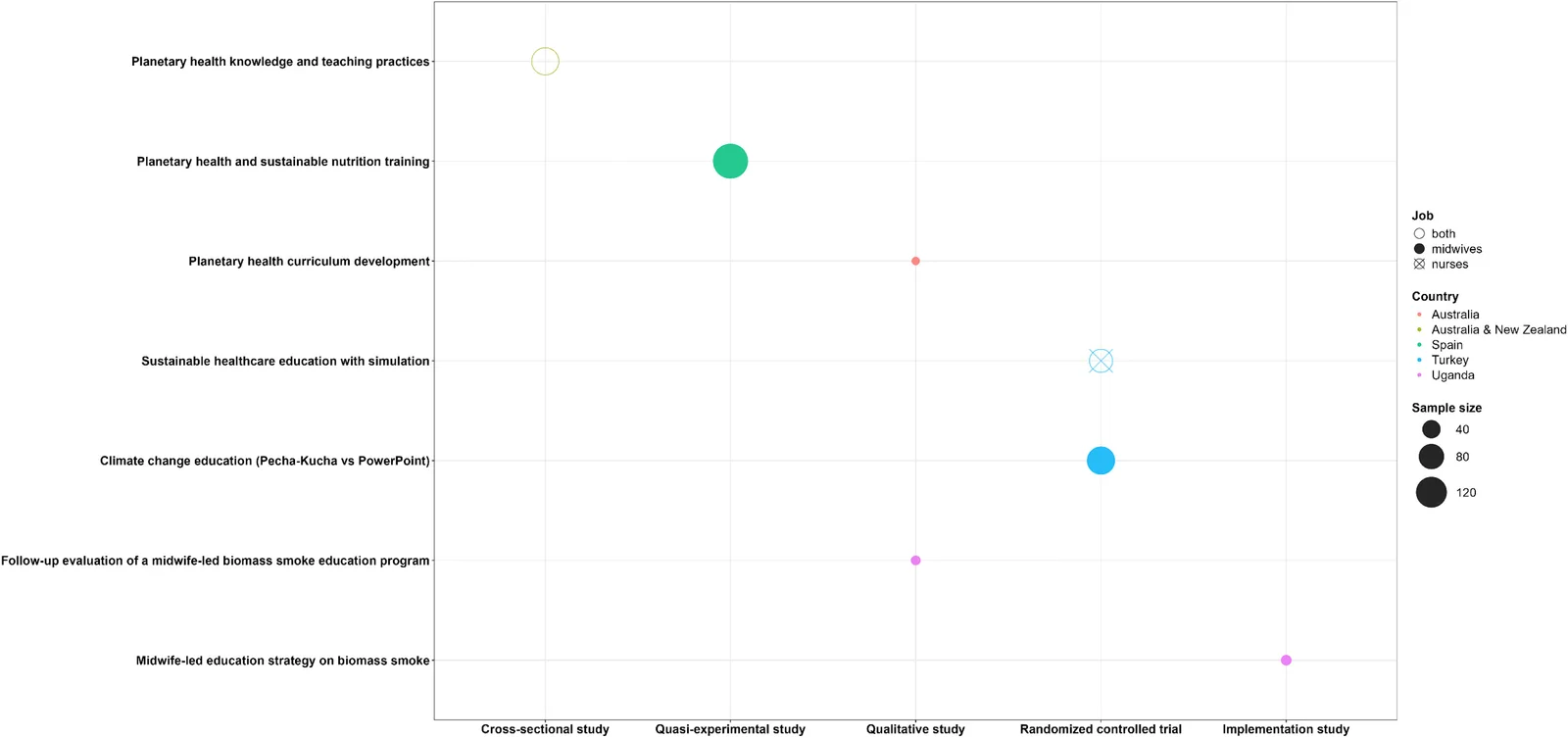
Evidence‑map bubble chart summarizing the seven included studies by type of education/training intervention, study design, sample size (bubble size), country (bubble color), and participant occupation (bubble shape).

Reported outcomes were concentrated mainly in knowledge and attitudinal domains, with fewer studies reporting confidence, behavioral, or system- and curriculum-level outcomes. No included study measured direct maternal, neonatal, or child clinical endpoints (**Supplementary Table 4**), which presents the synthesis of planetary health education and training outcomes for nurses and midwives across the knowledge, attitudes/acceptability, confidence/self-efficacy, practice-related, and curriculum/system-level domains, organized by review objective. Fig. 3 maps included studies by intervention characteristics, study design, sample size, country, participant occupation etc. Findings are synthesized below according to the seven review objectives. **Supplementary Figure 1** describes the main outcome domains (i.e., knowledge, attitudes, confidence/self‑efficacy, behavioral practice, and clinical/system‑level outcomes), together with their reported relevance to maternal and child health.

#### 3.2.1 Planetary health-related education and training initiatives

Across the seven included studies, planetary health-related education and training initiatives ranged from single online courses and simulation-based modules to curriculum validation exercises and community health worker-led programs. The target participants included nursing and midwifery students, practicing midwives, educators, and community members. Delivery approaches included cooperative simulation (İlaslan and Orak, 2025), the Pecha-Kucha method compared with conventional PowerPoint teaching (Kırca and Daglı, 2025), a 20-hour online course (Cabedo-Ferreiro et al., 2026), workshop-based curriculum validation (Catling et al., 2025), and community education sessions delivered by midwives or village health teams (Cartwright et al., 2022; Nantanda et al., 2019). This diversity suggests that planetary health education remains at an early and exploratory stage. The initiatives were independently developed across four countries, alongside one multi-country study, as presented in Table 1. No common or standardized curricular framework was evident across the included studies.

#### 3.2.2 Knowledge outcomes

Six of the seven included studies reported knowledge-related outcomes, with generally positive findings following structured educational interventions. Cabedo-Ferreiro et al. (2026) reported that 67% of participants passed the final course assessment. İlaslan and Orak (2025) found that sustainable healthcare knowledge at follow-up was significantly higher in the intervention group than in the control group. Similarly, Kırca and Daglı (2025) reported significantly higher post-training climate change awareness in the Pecha-Kucha group than in the PowerPoint group (19.27 ± 1.43 vs 13.75 ± 2.38). Nantanda et al. (2019) found that midwives’ mean knowledge scores increased from 65.5% before training to 85.5% after training, alongside similar improvements among participating women. Cartwright et al. (2022) also reported sustained understanding of the health risks associated with biomass smoke at follow-up. In contrast, Bonnamy et al. (2025) identified notable knowledge gaps among educators. Although 86.6% agreed that they could identify examples of planetary health concepts, 51.5% reported that they did not know how to explain these concepts to students.

Knowledge outcomes were assessed using heterogeneous methods, including course assessments, validated scales, and self-reported measures, which precluded meaningful pooled comparison across the studies.

#### 3.2.3 Attitudes or acceptability

Attitudinal outcomes were reported in five studies and were consistently positive, although they were assessed different, largely unstandardized tools. İlaslan and Orak (2025) found significantly higher sustainability attitude and motivation scores in the intervention group than the control group. Kırca and Daglı (2025) reported significantly higher satisfaction in the Pecha-Kucha group, with most students describing the session as clear, engaging, and enjoyable. Cabedo-Ferreiro et al. (2026) reported high course satisfaction, with 55.8% giving the maximum score. Bonnamy et al. (2025) found that educators generally held positive views toward integrating planetary health into curricula, despite their limited knowledge of the topic. Cartwright et al. (2022) reported that village health teams continued delivering the biomass smoke education program after the original project ended, suggesting sustained acceptability and feasibility beyond the funded intervention period.

#### 3.2.4 Confidence or self-efficacy

Confidence or self-efficacy was the least frequent and most narrowly measured outcome. Only Bonnamy et al. (2025) used a construct matching this domain, reporting low educator confidence in explaining planetary health to students. No other included studies used a validated confidence or self-efficacy measure. Cabedo-Ferreiro et al. (2026) reported “perceived applicability of acquired knowledge to professional practice”; this construct reflects a self-reported practice-related outcome rather than confidence or self-efficacy, and is synthesized under practice-related changes below.

#### 3.2.5 Practice-related changes

Practice-related outcomes were reported in four studies, with the strongest evidence from the two Ugandan biomass smoke education programs. Nantanda et al. (2019) reported behavioral changes among midwives and women, including avoiding smoke while cooking, using dry wood, using solar lighting, and improving ventilation. Cartwright et al. (2022) similarly reported household and cooking-related behavior changes — reconstructing cooking areas, improving ventilation, installing chimneys, and switching from kerosene or candles to solar lighting — and found that 11 of 12 mothers had shared the education with others; perceived health improvements such as reduced coughing were also reported but were self-reported and not clinically verified. İlaslan and Orak (2025) found significantly better simulated sustainable care skill performance in the intervention group, with correct waste separation observed in 77.77% of the intervention group compared with 0% and 11.11% across the two control comparisons. Cabedo-Ferreiro et al. (2026) reported perceived applicability of acquired knowledge to practice (71.6% giving the maximum score) but did not measure an objective practice or behavior change. Across these four studies, practice-related evidence relied on self-report or simulated performance rather than objectively verified clinical behavior.

#### 3.2.6 Curriculum or system-level outcomes

Curriculum- or system-level outcomes were reported in three studies. Catling et al. (2025) validated planetary health knowledge and skill statements for undergraduate midwifery curricula; most statements were rated important or highly important by midwifery academics, and only minimal revisions were required. Nantanda et al. (2019) reported that education materials were co-developed, serially tested, and formally approved by Uganda's Ministry of Health before implementation. Bonnamy et al. (2025) while not itself a curriculum intervention, found that most educators had not previously taught planetary health content, indicating a system-level capacity gap relevant to future curriculum integration.

#### 3.2.7 Maternal and child health relevance

No included study measured direct maternal, neonatal, or child clinical endpoints such as preterm birth, birth weight, breastfeeding duration, neonatal morbidity, or child respiratory illness. Two studies demonstrated practice-related relevance: the Ugandan biomass smoke education programs (Cartwright et al., 2022; Nantanda et al., 2019) reported behavioral changes among mothers and pregnant women intended to reduce household air pollution exposure for mothers, infants, and children. Cabedo-Ferreiro et al. (2026) addressed breastfeeding and sustainable maternal nutrition content but measured knowledge, satisfaction, and perceived applicability rather than actual breastfeeding behavior, maternal diet, or infant and child health outcomes. The remaining studies had indirect maternal and child health relevance through distinct pathways: Bonnamy et al. (2025) through educator preparedness to address climate- and environment-related risks in maternal, neonatal, and child health care; Catling et al. (2025) through curriculum strengthening intended to prepare future midwives to address environmental determinants of pregnancy, childbirth, and newborn health; and İlaslan and Orak (2025) and Kırca and Daglı (2025) through building sustainable-practice knowledge, attitudes, and skills among nursing and midwifery students who will provide future maternal and child care.

## 4 Discussion

To our knowledge, this is the first scoping review to map planetary health-related education and training initiatives for nurses and midwives, with specific attention to their relevance for maternal and child health. The review did not identify evidence of direct maternal, neonatal, or child clinical impacts; instead, it captured how educational interventions may influence maternal and child health indirectly, through workforce preparedness, educator capacity, curriculum development, knowledge acquisition, and practice-related behavioral change. The seven included studies indicate that this is an emerging field, geographically narrow in scope, with a disconnect between the stated ambition for curricular integration and the evidence currently available on the effectiveness of these interventions (Carrion et al., 2025).

### 4.1 Major findings

#### 4.1.1 Scope and educational approaches

The seven included studies varied considerably in the scope and focus of the planetary health content they addressed, ranging from climate change awareness (Kırca and Daglı, 2025) and sustainable healthcare (İlaslan and Orak, 2025) to biomass smoke exposure (Cartwright et al., 2022; Nantanda et al., 2019), sustainable maternal nutrition (Cabedo-Ferreiro et al., 2026), curriculum-based competencies (Catling et al., 2025), and educator knowledge and teaching practices (Bonnamy et al., 2025). This variability is better understood as a difference in scope rather than conceptual inconsistency, and is consistent with the broader planetary health education literature: Carrion et al. (2025) identified substantial heterogeneity in frameworks, competencies, content, and teaching approaches across the health professions, with no single standardized model yet established. In terms of educational approach, interactive and applied teaching methods appeared to yield more favorable outcomes than passive instruction (Selvam et al., 2024). The Turkish randomized studies demonstrated stronger results with simulation-based sustainable healthcare education and the Pecha-Kucha method for climate change awareness (Kırca and Daglı, 2025), while the Ugandan studies showed that midwife-led, community-based biomass smoke education extended beyond knowledge gain to reported behavioral change among women (Cartwright et al., 2022; Nantanda et al., 2019). These findings align with the wider literature emphasizing experiential, participatory, and practice-oriented learning in planetary health education over traditional didactic teaching (Dogan et al., 2025; Selvam et al., 2024), which is particularly relevant for nursing and midwifery given the applied, communication- and community-oriented nature of professional practice.

#### 4.1.2 Knowledge outcomes

Reported outcomes were concentrated overwhelmingly in the domains of knowledge and, to a lesser extent, attitudes, while confidence, behavioral change, and system-level outcomes were assessed far less frequently. This pattern is consistent with the wider literature on nursing and health professional education, where the integration of planetary health and climate-related content has increased, but evaluation has remained largely focused on awareness, perceptions, and short-term learning outcomes rather than sustained professional practice or patient-level effects (Dogan et al., 2025; Roberge et al., 2025; Tiitta et al., 2024). The strong emphasis on knowledge-based outcomes likely reflects the early developmental stage of planetary health education within nursing and midwifery: when emerging topics are first incorporated into curricula, evaluation tends to focus on immediate indicators such as participant satisfaction, comprehension, and short-term awareness gains (Selvam et al., 2024).

#### 4.1.3 Attitudes and acceptability

Attitudinal outcomes were reported alongside knowledge outcomes in most studies and generally showed favorable shifts in participants’ recognition of planetary health as relevant to maternal and child healthcare. Among educators, the survey included in this review also found broadly positive attitudes towards integrating planetary health into nursing and midwifery curricula (Bonnamy et al., 2025). This suggests that acceptance of the topic among learners and faculty may not be the primary barrier to curricular integration. Rather, as discussed below, the main challenge lies in translating this acceptance into confidence and applied competence.

#### 4.1.4 Confidence and self-efficacy

For planetary health education to meaningfully affect maternal and child health, future evaluation should extend beyond satisfaction and awareness to examine whether nurses and midwives develop and maintain confidence and self-efficacy in counselling pregnant women on climate- and environment-related risks — for example using validated self-efficacy instruments, repeated follow-up assessment, or observed counselling performance. This recommendation is consistent with Attawet et al. (2025), who reported variability in midwives' preparedness to address climate-related maternal and child health challenges, and with the survey of nursing and midwifery educators included in this review. Despite broad support for integrating planetary health into curricula, confidence in actually teaching the topic remained limited, with over half (51.5%) of educators reporting that they did not know how to explain planetary health concepts to students, and most having not previously taught planetary health content (Bonnamy et al., 2025). This confidence gap mirrors findings from recent reviews in medical and nursing education, which have consistently identified inadequate faculty expertise, limited curricular time, and insufficient institutional support as key barriers to meaningful integration (Roberge et al., 2025; Selvam et al., 2024). Together, these findings indicate that the gap is not simply one of curricular omission but of educator and learner readiness: without sustained faculty development and system-level implementation capacity, planetary health education risks remaining fragmented and dependent on individual initiative rather than embedded within routine maternal and child health workforce preparation.

#### 4.1.5 Practice-related change

Evidence of practice-level or behavioral change was limited to two of the seven included studies. The Ugandan biomass-smoke interventions, delivered by midwives at community level, reported behavioral change and perceived health improvements among women following the intervention (Cartwright et al., 2022; Nantanda et al., 2019), whereas the remaining five studies did not extend measurement beyond knowledge, attitudes, or educator/curriculum-level outcomes. This scarcity of practice-level evidence indicates that, although planetary health content can shift what nurses and midwives know and believe, whether this translates into changed clinical or counselling behavior remains largely untested.

#### 4.1.6 Curriculum and system-level outcomes

System-level evidence was similarly sparse. Catling et al. (2025) highlighted the need for curriculum validation and integration processes, while Bonnamy et al. (2025) identified educator knowledge and confidence gaps that constrain implementation at the institutional level. None of the included studies directly evaluated institutional support or policy integration; however, taken together with findings from prior reviews (Attawet et al., 2025; Roberge et al., 2025), these observations suggest that inadequate training opportunities, limited institutional commitment, and minimal incorporation into accreditation or policy frameworks may all constrain the implementation of planetary health education in nursing and midwifery.

#### 4.1.7 Maternal and child health relevance

Across all seven studies, the relevance of planetary health education to maternal and child health was indirect rather than direct. No study reported maternal, neonatal, or child clinical endpoints such as preterm birth, birth weight, or neonatal morbidity. Instead, relevance was established through workforce preparedness, educator capacity, curriculum development, knowledge acquisition, and, in two studies, reported behavioral change (Attawet et al., 2025; Bonnamy et al., 2025). This is an important finding in itself: it indicates that the field has yet to demonstrate whether improving nurses' and midwives' planetary health knowledge and confidence translates into measurable benefit for mothers, neonates, or children.

### 4.2 Geographic and equity considerations

The concentration of evidence in relatively better-resourced settings is consistent with broader planetary health education literature. Carrion et al. (2025) global scoping review found major heterogeneity in frameworks, competencies, content, and teaching approaches, and reported that most educational activity has been generated in high-income contexts rather than settings with the greatest climate-related health burden. This geographical mismatch is especially important in the present review because maternal and child health risks from heat, air pollution, food insecurity, and environmental disruption are often most severe in low- and middle-income countries (Prag et al., 2026; Raghavan et al., 2025). The two clearest gaps identified by this review is both the paucity of published studies overall, and the lack of context-specific evidence from regions where climate-sensitive maternal and child health risks are greatest (Attawet et al., 2025).

### 4.3 Implications for nursing and midwifery education

Building on the findings above, four specific implications follow for nursing and midwifery education. First, because interactive and applied teaching methods were associated with stronger outcomes than passive instruction, curricula should prioritize simulation-based learning, the Pecha-Kucha method, and community-based, practice-oriented formats over lecture-based delivery (Kırca and Daglı, 2025; Cartwright et al., 2022; Nantanda et al., 2019). Second, because educator confidence rather than acceptability emerged as the principal barrier to integration, faculty development should be prioritized to build educators' capacity to explain and teach planetary health content, directly addressing the gaps identified by Bonnamy et al. (2025). Third, because no included study measured maternal, neonatal, or child clinical outcomes, curricula and evaluation frameworks should build in mechanisms to track downstream practice change, such as structured clinical placements or competency assessments linked to maternal and child health counselling. Fourth, given the documented gaps in institutional support and policy integration (Catling et al., 2025; Bonnamy et al., 2025), planetary health content should be embedded longitudinally across pre-service curricula and continuing professional education, and explicitly incorporated into accreditation and competency frameworks (Jacobsen et al., 2024; Roberge et al., 2025), rather than delivered as isolated or elective activities. For patient care, nurses and midwives are uniquely positioned to counsel pregnant women and families on environmental exposures affecting pregnancy outcomes, neonatal health, and child development (World Health Organization, 2021). Strengthening these competencies has the potential to improve climate-sensitive maternal and child health outcomes, particularly in low- and middle-income settings where such risks are greatest (Attawet et al., 2025; Roberge et al., 2025).

### 4.4 Research gaps and future directions

This review identified several important gaps in the current evidence base. First, the literature remains limited in volume and geographically uneven. Although four of the seven included studies were conducted in middle-income countries, specifically Uganda and Turkey (Cartwright et al., 2022; İlaslan and Orak, 2025; Kırca and Daglı, 2025; Nantanda et al., 2019), evidence remains absent from many regions that experience a substantial burden of climate-sensitive maternal and child health risks, including large parts of Africa, South Asia, and Latin America. Therefore, the available evidence cannot yet be considered geographically representative (2.3). As detailed in the major findings above (Section 4.1), the included studies also assessed short-term outcomes far more often than confidence, practice-level, or system-level outcomes, and none directly evaluated maternal, neonatal, or child clinical indicators; this evaluation gap represents a priority for future research and is therefore not restated here. Future research should prioritize context-specific studies in resource-constrained settings, adopt longitudinal and mixed-methods designs, and assess whether educational interventions lead to sustained competency development and measurable practice change — for example using validated self-efficacy instruments, repeated follow-up assessments, observed counselling performance, or competency-based evaluations conducted over time. Greater attention is also needed to faculty development, implementation processes, and the integration of planetary health into accreditation and competency frameworks. Establishing more standardized conceptual and evaluative approaches will be essential to strengthen the field and support meaningful translation into maternal and child health practice. As a future perspective, we suggest the development of a standardized Planetary Health Literacy Score or similar metric to evaluate competencies among health professionals. Such a tool could help measure the effectiveness of educational interventions and facilitate comparisons across professional groups and contexts. The key messages from our study are presented in Table 2. To synthesize the broader implications of these findings, a SWOT framework is presented in **Supplementary Figure 2**, summarizing the strengths, weaknesses, opportunities, and threats identified for the future development of planetary health education in nursing and midwifery.

**Table 2:** Key messages from this study.

• Evidence on planetary health education for nurses and midwives is nascent, with only seven peer-reviewed primary studies published between 2019 and 2026.
• Knowledge gains are the most consistently reported educational outcome; evidence of sustained practice change and clinical impact is sparse.
• Community-based midwife-led biomass smoke education programs in Uganda offer the most direct evidence linking midwife training to maternal behavior change relevant to pregnancy outcomes.
• Evidence from one included study suggests that nursing and midwifery educators may have limited knowledge and low confidence in explaining planetary health concepts to students, which could hinder curriculum integration.
• Evidence from low- and middle-income countries-where planetary health risks and maternal vulnerabilities converge most acutely-is critically underrepresented.
• Embedding standardized planetary health competencies within professional accreditation frameworks is essential for sustainable workforce transformation.

### 4.5 Strengths and limitations

The use of established scoping review methodology enhances transparency and breadth. However, this scoping review has several limitations. First, the small number of included studies (n = 7) limits the generalizability of our findings, indicating that the field is in its infancy. However, the objective of a scoping review is to map the extent, nature, and characteristics of available evidence rather than to provide statistically representative estimates of a global phenomenon. The identification of only seven eligible studies despite a comprehensive search across multiple databases is itself an important finding, highlighting the limited and emerging nature of the evidence base on planetary health education for nurses and midwives worldwide (Arksey and O'malley, 2005; Tricco et al., 2018). Second, as a scoping review, it did not include a formal quality appraisal, meaning our conclusions are based on the available evidence without a formal assessment of study quality or risk of bias. Third, by limiting the search to English-language publications, relevant studies in other languages may have been missed.

## 5 Conclusion

The main contribution of this scoping review is its demonstration that the field of planetary health education has moved beyond conceptual advocacy for healthcare workforce but has yet to generate a sufficiently rigorous evidence base to guide maternal and child health relevant nursing and midwifery practice. Existing evidence remains fragmented, is predominantly centered on short-term educational outcomes, and is only weakly linked to measurable maternal and child health indicators (Dogan et al., 2025; Vandenberg et al., 2025). Important gaps persist in low- and middle-income countries focused research, faculty preparedness, and evaluation of system- and patient-level outcomes. By mapping the current evidence and identifying these limitations, this review provides a foundation for more rigorous, context-sensitive, and practice-oriented research to strengthen the contribution of nurses and midwives to maternal and child health in the context of planetary change.

**Appendix A.** Preferred Reporting Items for Systematic reviews and Meta-Analyses extension for Scoping Reviews -Fillable-Checklist

**Appendix B.** Supplementary files

## Declaration of generative AI and AI-assisted technologies in the manuscript preparation process

During the preparation of this work the authors used Claude 5 Sonnet in order to check the grammar, language and coherence. After using this tool, the authors reviewed and edited the content as needed and take full responsibility for the content of the published article.

## Funding

The authors received no financial support for the research.

## Data availability

No primary data was used for the research described in the article.

## Ethics approval and consent to participate

Not applicable.

## Statistics statement

The authors have checked to make sure that the submission conforms as applicable to the Journal’s statistical guidelines.

## Funding

This research did not receive any specific grant from funding agencies in the public, commercial, or not-for-profit sectors.

## CRediT authorship contribution statement

**Sadia Afrin:** Writing – original draft, Software, Methodology, Investigation, Formal analysis, Data curation, Conceptualization. **Pascal Petit:** Writing – review & editing, Visualization, Validation, Software, Methodology, Formal analysis. **Nicolas Vuillerme:** Writing – review & editing, Validation, Resources. **Muhammad Asaduzzaman:** Writing – review & editing, Writing – original draft, Validation, Supervision, Resources, Project administration, Methodology, Conceptualization.

## Declaration of competing interest

The authors declare that they have no known competing financial interests or personal relationships that could have appeared to influence the work reported in this paper.
