# Supplementary material for: Planetary health in practice: A scoping review of education and training for nurses and midwives
Source: Int J Nurs Stud Adv. 2026 Sep 10;11:100670. doi: 10.1016/j.ijnsa.2026.100670 (PMC13598162; doi:10.1016/j.ijnsa.2026.100670)
Supplement: Supplementary file 2 [file mmc2.docx]

**Supplementary Information**

**Planetary Health in Practice: A Scoping Review of Education and Training for Nurses and Midwives**

**Tables**

**Supplementary Table 1: Key terms for developing the comprehensive search strategy**

| **Population** | **Content** | **Context** |
| --- | --- | --- |
| Midwives | Education curriculum | Planetary health |
| Nurse | Training program | Maternal health |
| Nursing student | Educational program | Child health |
| Midwifery students | Education |  |
| Midwifery educator | Teaching |  |
| Nurse educator |  |  |

**Supplementary Table 2: Comprehensive search strategies and number of records retrieved from PubMed, EMBASE, and Scopus**

| **Serial No** | **Search queries** | **Search Result** |
| --- | --- | --- |
| #1 Planetary health | "Planetary health"[All Fields] | 4,634 |
| #2  Education / teaching | "educates"[All Fields] OR "education"[MeSH Subheading] OR "education"[All Fields] OR "education"[MeSH Terms] OR "education s"[All Fields] OR "educational"[All Fields] OR "educative"[All Fields] OR "teaching"[MeSH Terms] OR "teaching"[All Fields] OR "educate"[All Fields] OR "educated"[All Fields] OR "educating"[All Fields] OR "educations"[All Fields] | 2,782,934 |
| #3= #1 AND #2  Planetary health AND education | "Planetary health"[All Fields] AND ("educates"[All Fields] OR "education"[MeSH Subheading] OR "education"[All Fields] OR "education"[MeSH Terms] OR "education s"[All Fields] OR "educational"[All Fields] OR "educative"[All Fields] OR "teaching"[MeSH Terms] OR "teaching"[All Fields] OR "educate"[All Fields] OR "educated"[All Fields] OR "educating"[All Fields] OR "educations"[All Fields]) | 988 |
| #4  Nursing / midwifery population | "nurs*"[All Fields] OR "midwiv*"[All Fields] | 1,314,550 |
| #5= #3 AND #4  Final PubMed search combination | "Planetary health"[All Fields] AND ("educates"[All Fields] OR "education"[MeSH Subheading] OR "education"[All Fields] OR "education"[MeSH Terms] OR "education s"[All Fields] OR "educational"[All Fields] OR "educative"[All Fields] OR "teaching"[MeSH Terms] OR "teaching"[All Fields] OR "educate"[All Fields] OR "educated"[All Fields] OR "educating"[All Fields] OR "educations"[All Fields]) AND ("nurs*"[All Fields] OR "midwiv*"[All Fields]) | 143 |

MeSH: Medical Subject Heading

Filter by date: From 1st January 2015 to 31st January 2026

Search result: 142 results

Search date: 31-01-2026

**EMBASE**

| **Serial No** | **Search queries** | **Search Result** |
| --- | --- | --- |
| #1 | 'nurse' |  |
| #2 | 'midwife' |  |
| #3 | 'training' |  |
| #4 | 'education' |  |
| #5= #2 OR #3 | #2 OR #3 |  |
| #6= #4 OR #5 | #4 OR #5 |  |
| #7 | 'planetary health':ab,ti |  |
| #8= #5 AND #6 AND #7  Final EMBASE search combination | #5 AND #6 AND #7 | 41 |

Filter by date: From 1st January 2015 to 31st January 2026

Search result: 41 results

Search date: 31-01-2026

**Scopus**

| **Serial No** | **Search queries** | **Search Result** |
| --- | --- | --- |
| #1  Final Scopus search combination | (TITLE-ABS-KEY (Planetary Health) AND TITLE-ABS-KEY (education) OR TITLE-ABS-KEY (training) AND TITLE-ABS-KEY (Nurse) OR TITLE-ABS-KEY (midwifery)) | 75 |

Filter by date: From 1st January 2015 to 31st January 2026

Search result: 75 results

Search date: 31-01-2026

**Supplementary Table 3: Included articles**

| **Serial No** | **Author’s name and publication year** | **Title** |
| --- | --- | --- |
| 1 | Bonnamy et al., 2024 | Australian and New Zealand nursing and midwifery educators' planetary health knowledge, views, confidence and teaching practices |
| 2 | Cabedo-Ferreiro et al., 2026 | The role of midwives in advancing planetary health: Insights from the GREEN MOTHER project in Spain |
| 3 | Cartwright et al.,2022 | Perceptions of long-term impact and change following a midwife-led biomass smoke education program for mothers in rural Uganda |
| 4 | Catling et al.,2025 | Enhancing midwifery course curricula: validating knowledge and skills in climate change and planetary health |
| 5 | Ilaslan et al.,2025 | Sustainable healthcare education using cooperative simulation in developing nursing students’ knowledge, attitude and skills: A randomized controlled study |
| 6 | Kırca et al., 2025 | Evaluation of the effectiveness of education given to midwifery students with The Pecha Kucha Method on climate change awareness |
| 7 | Nantanda et al., 2019 | Feasibility and acceptability of a midwife-led health education strategy to reduce exposure to biomass smoke among pregnant women in Uganda |

**Supplementary Table 4:** **Synthesis of Planetary Health Education and Training Outcomes for Nurses and Midwives, by Review Objective**

| **Author, year** | **Education / training initiative** | **Knowledge** | **Attitudes / acceptability** | **Confidence / self-efficacy** | **Practice-related changes** | **Curriculum / system-level outcomes** | **Maternal and child health relevance** |
| --- | --- | --- | --- | --- | --- | --- | --- |
| Bonnamy et al., 2025 | Online cross-sectional survey of nursing & midwifery educators' planetary health knowledge, views, confidence, and teaching practices. | Limited understanding beyond climate change; most had not previously taught planetary health content. | Generally positive views toward integrating planetary health into curricula. | Low confidence explaining planetary health; 51.5% did not feel able to explain concepts despite 86.6% recognizing examples. | Not applicable (cross-sectional survey; no intervention or practice change assessed). | Findings indicate limited educator readiness, a capacity gap relevant to future curriculum integration. | Indirect. No maternal, neonatal, or child clinical outcome measured; relevance is through educator preparedness to address climate- and environment-related risks in MNCH care. |
| Cabedo-Ferreiro et al., 2026 | 20-hour online course for healthcare professionals (predominantly midwives) on breastfeeding, sustainable maternal nutrition, and planetary health. | 67% of participants passed the final course assessment. | High satisfaction; 55.8% gave the highest overall satisfaction score. | Not measured using a validated confidence/self-efficacy scale. | Perceived applicability of acquired knowledge to practice (71.6% gave the maximum score); no objective behavior changes measured. | Not measured. | Indirect. Addressed breastfeeding and sustainable maternal nutrition, but measured knowledge/satisfaction/perceived applicability rather than actual breastfeeding, diet, or child health outcomes. |
| Catling et al., 2025 | Validation of planetary health, climate change, and sustainability curriculum domains for undergraduate midwifery education. | Not applicable (validators were midwifery academics; no learner knowledge gain measured). | Not measured among learners. | Not measured. | Not measured. | Content and face validity established; most statements rated important/highly important; minimal revisions required. | Indirect. Curriculum strengthening intended to prepare future midwives to address environmental determinants of pregnancy, childbirth, and newborn health. |
| İlaslan & Şahin Orak, 2025 | Sustainable healthcare education using cooperative learning and high-fidelity simulation for nursing students (RCT vs self-directed learning). | Significantly higher knowledge at follow-up in the intervention group. | Significantly higher sustainability attitude and motivation scores in the intervention group. | Not measured using a validated scale. | Significantly better simulated sustainable care skills; correct waste separation in 77.77% of the intervention group vs 0%/11.11% in comparison groups. | Not measured. | Indirect. Sustainable nursing practices may reduce environmental harm in healthcare settings, including maternal and neonatal care environments. |
| Kırca & Daglı, 2025 | Climate change education for midwifery students via Pecha-Kucha vs PowerPoint (RCT). | Significantly higher post-training climate change awareness in the Pecha-Kucha group (19.27±1.43 vs 13.75±2.38). | Significantly higher satisfaction in the Pecha-Kucha group; positive feedback on clarity, interest, and engagement. | Not measured. | Not measured. | Not measured. | Indirect. Addressed climate awareness among future midwives; did not measure practice change or maternal/neonatal/child health outcomes. |
| Cartwright et al., 2022 | Qualitative follow-up of a midwife-led biomass smoke education program for mothers in rural Uganda. | Sustained understanding of biomass smoke risks reported at follow-up. | Continued program delivery after project ended, suggesting sustained acceptability and feasibility. | Not measured. | Household/cooking behavior changes (ventilation, chimneys, solar lighting); 11/12 mothers shared knowledge with others; perceived health improvements self-reported, not clinically verified. | Not measured. | Indirect. Maternal household behavior changes reported that may reduce household air pollution exposure for pregnant women, mothers, infants, and children; not objectively verified clinically. |
| Nantanda et al., 2019 | Midwife-led biomass smoke education strategy for pregnant and postnatal women in Uganda. | Midwives' average knowledge increased from 65.5% to 85.5%; women's knowledge improved across several items. | Feasibility/acceptability of midwife-led delivery reported; 12 midwives and 40 village health team members sensitized, 244 women educated. | Not measured. | Reported behavioral changes/intentions: avoiding smoke while cooking, using dry wood, solar lighting, improved ventilation. | Education materials co-developed, serially tested, and approved by the Ministry of Health before implementation. | Indirect. Intervention targeted pregnant/postnatal women to reduce biomass smoke exposure for mothers, unborn babies, and children; no direct clinical outcomes measured. |

**Supplementary Figure 1**. Heatmap presenting the main outcome domains (i.e., knowledge, attitudes, confidence/self‑efficacy, behavioral practice, and clinical/system‑level outcomes), together with their reported relevance to maternal and child health.

**Supplementary Figure 2**. SWOT analysis of planetary health education and training for nurses and midwives.

Diagram summarizing key Strengths, Weaknesses, Opportunities, and Threats identified for the future development of planetary health education in maternal and child health contexts.
